# Case Report: A rare case of asymptomatic unroofed coronary sinus in a dog: diagnostic imaging and genetic findings

**DOI:** 10.3389/fvets.2025.1611021

**Published:** 2025-07-30

**Authors:** Yoonju Choi, Wonkyoung Yoon, Byung-Yong Park, Young-Jin Jang, Kichang Lee, Hakyoung Yoon

**Affiliations:** ^1^Department of Veterinary Medical Imaging, College of Veterinary Medicine, Jeonbuk National University, Iksan, Republic of Korea; ^2^Guardian Angel Veterinary Hospital, Anyang, Republic of Korea; ^3^Department of Veterinary Embryology, College of Veterinary Medicine, Jeonbuk National University, Iksan, Republic of Korea; ^4^Center for Large Animals Convergence Research, Korea Institute of Toxicology, Jeongeup, Republic of Korea

**Keywords:** canine, congenital heart disease, atrial septal defect, case report, echocardiography, computed tomography, genetic variant

## Abstract

A 2-year-old, 3.8 kg spayed female Pomeranian was presented for a routine health examination with no clinical signs. Physical examination revealed no cardiac murmur. Blood tests were unremarkable and electrocardiography revealed normal sinus rhythm. Thoracic radiography showed a normal cardiac silhouette. Transthoracic echocardiography identified a left atrial size at the upper limit of the normal range (left atrium-to-aortic root ratio: 1.7) and a markedly visualized tubular structure along the posterior wall of the left atrium, presumed to be the coronary sinus. Color Doppler imaging revealed continuous flow into the right atrium without evidence of atrial septal defect. There was no evidence of pulmonary hypertension and a bubble study excluded an intracardiac right-to-left shunt. Subsequent computed tomography identified a 3.6 mm partial defect between the midportion of the coronary sinus and the left atrium, consistent with a partial unroofed coronary sinus. Despite the absence of coronary sinus dilation, contrast enhancement patterns were similar in both the coronary sinus and the left atrium, supporting the presence of a left-to-right shunt. No other congenital abnormalities were identified, and regular follow-up with echocardiography and clinical sign monitoring was recommended. Whole-exome sequencing revealed four unique missense single nucleotide variants in genes potentially implicated in cardiac development. This case highlights that unroofed coronary sinus can occur without clinical signs or associated anomalies in dogs and further presents potential genetic variants identified through whole-exome sequencing, contributing to a better understanding of this rare defect.

## 1 Introduction

A cardiac shunt is a congenital heart defect characterized by an abnormal passage between two cardiac chambers or between distinct circulatory pathways, causing blood to bypass its normal route (1, 2). In dogs, congenital heart diseases such as pulmonic stenosis and aortic stenosis are relatively common (3, 4), whereas unroofed coronary sinus (UCS) is exceedingly rare, with only two veterinary cases reported to date (5, 6).

UCS involves partial or complete defect of the coronary sinus roof, resulting in an abnormal communication with the left atrium (LA). First described by Raghib et al. (7) in 1965, UCS is considered a rare form of atrial septal defect, which accounts for 0.1% of all congenital heart diseases in humans (8). Most human cases of UCS are incidentally detected in asymptomatic individuals (9). When not identified during childhood, the defect is often diagnosed later in adulthood, with one study reporting cases in individuals aged 30 to 56 years (10). Larger defects can lead to right ventricular volume overload and heart failure, necessitating surgical closure (8).

In veterinary medicine, asymptomatic UCS has not been previously documented, and the genetic factors underlying UCS in dogs remain unknown. Here, we report a case of an asymptomatic UCS in a dog, discovered incidentally during a routine health examination, and diagnosed using transthoracic echocardiography and computed tomography (CT). We also present potential genetic variants identified through whole-exome sequencing (WES), contributing to our understanding of this rare anomaly in dogs.

## 2 Case description

A 2-year-old spayed female Pomeranian dog weighing 3.8 kg was presented for a routine health examination with no history of cardiovascular disease or clinical signs. Vital signs were unremarkable, with a systolic blood pressure of 144 mmHg and a heart rate of 80 beats per minute. No cardiac murmur was detected. Blood tests were unremarkable, and electrocardiography (ECG) revealed a normal sinus rhythm.

**Figure 1:**
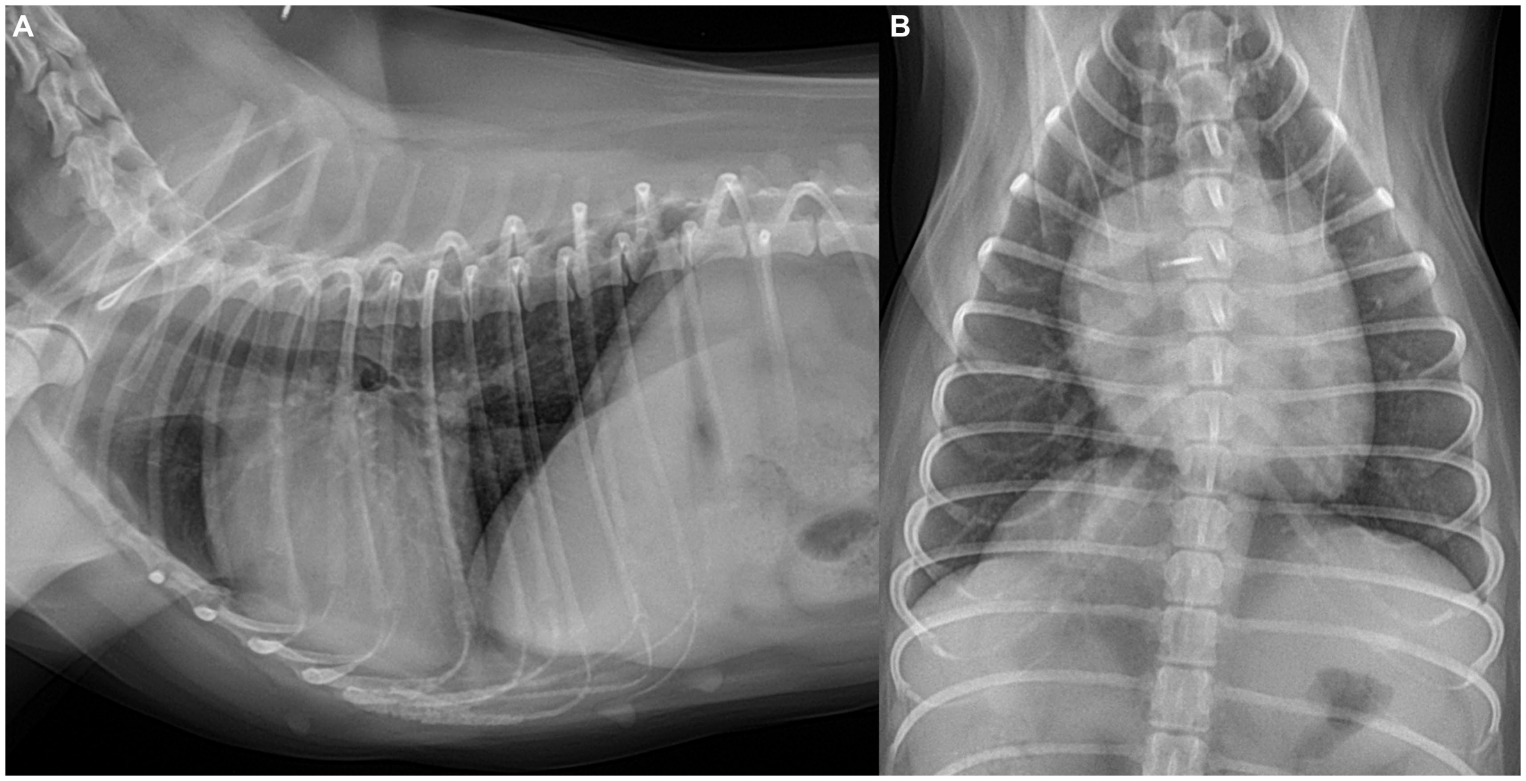
Thoracic radiographs of the dog. **(A)** Right lateral and **(B)** ventrodorsal views. Radiographs revealed a cardiac silhouette within normal limits, with the pulmonary vessels and caudal vena cava also measuring within normal limits.

Thoracic radiography (DRGEM, Seoul, Republic of Korea) demonstrated a cardiac silhouette within normal limits, with a vertebral heart size of 10.8 (reference range: 10.69 ± 0.62) and a vertebral left atrial size of 2.1 (reference range: 2.17 ± 0.26) (11). Pulmonary vessels appeared normal, as the cranial lobar pulmonary vessels measured less than 1.2 times the diameter of the proximal third of the fourth rib, and the caudal lobar vessels did not exceed the ninth rib thickness (12) (Figure 1).

**Figure 2:**
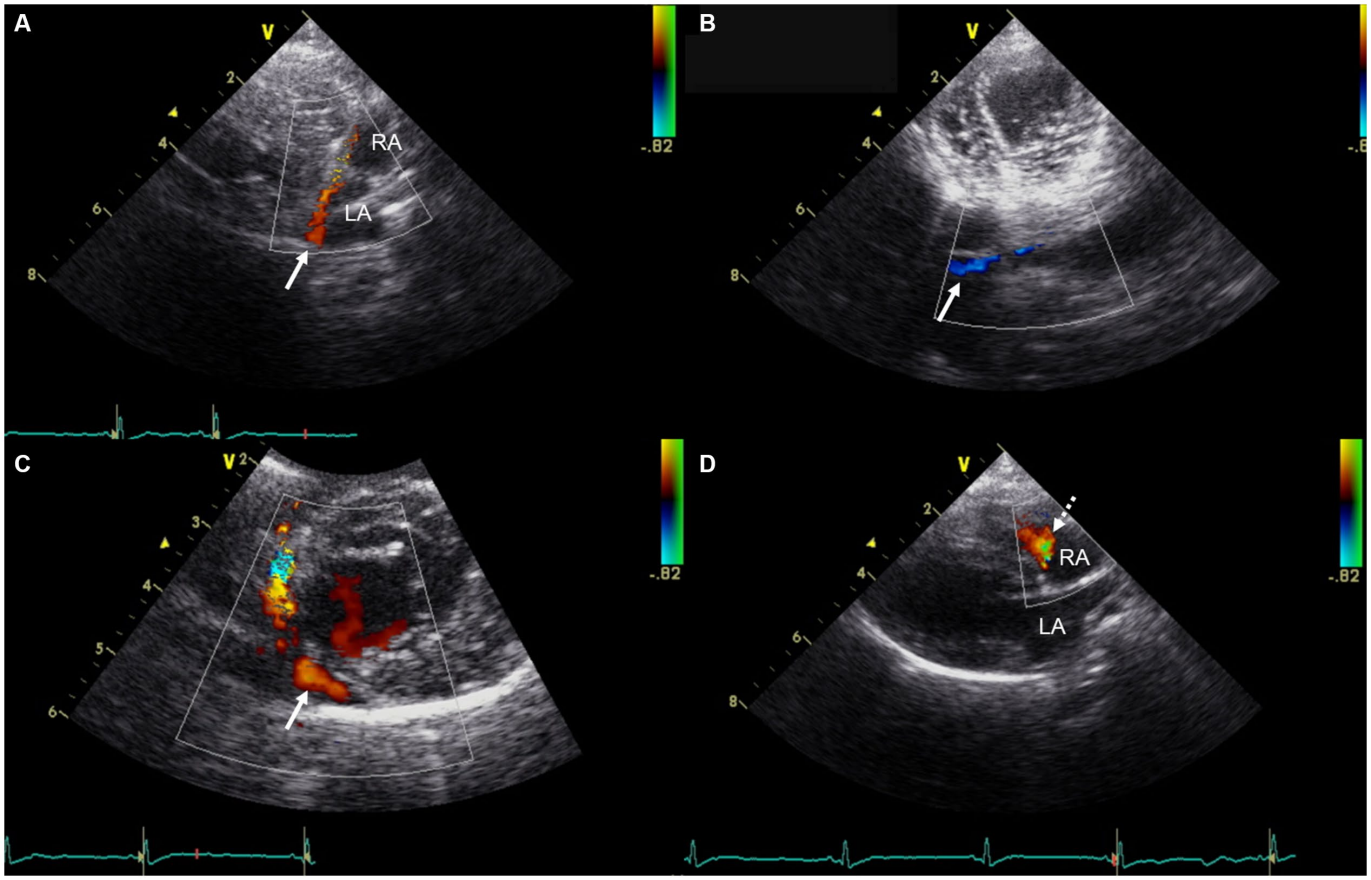
Transthoracic echocardiographic images showing a markedly visualized coronary sinus and continuous blood flow into the right atrium. **(A)** Modified right parasternal long-axis view. **(B)** Modified left apical four-chamber view at the level of the chordae tendineae. **(C)** Modified right parasternal short-axis view. **(D)** Right parasternal long-axis four-chamber view. In panels **(A–C)**, Color Doppler imaging identified a markedly visualized tubular structure (white arrow) running along the posterior aspect of the LA and extending caudally to the RA, near the tricuspid valve annulus, which is presumed to represent the coronary sinus. In panel **(D)**, Color Doppler imaging demonstrates continuous blood flow (white dashed arrow) of unidentified origin entering the RA. LA, left atrium; RA, right atrium.

Transthoracic echocardiography (GE Vivid 7, Horten, Norway) using a 5-MHz transducer identified a LA size at the upper limit of the normal range, with a left atrium-to-aortic root ratio of 1.7 (upper reference limit: 1.62–1.83) (13). The left ventricle, right atrium (RA) and right ventricle appeared normal in size. There was no evidence of pulmonary hypertension, systolic dysfunction, or diastolic dysfunction. Color Doppler imaging identified a markedly visualized tubular structure running along the posterior aspect of the LA and extending caudally to the RA near the tricuspid valve annulus, which was presumed to be the coronary sinus. Color Doppler imaging demonstrated a continuous blood flow of unclear origin into the RA, with a velocity of 0.5 m/s (Figure 2). No atrial septal defect was identified. The pulmonary-to-systemic blood flow ratio (*Q*_p_/*Q*_s_) was 1.3 (reference range: 0.71–1.29) (14). Contrast-enhanced echocardiography using hand-agitated saline confirmed the absence of an intracardiac right-to-left shunt. As the origin of the continuous blood flow into the right atrium was not clearly identified on echocardiography, additional CT (SIEMENS Healthineers, Erlangen, Germany) was performed to differentiate potential congenital cardiac anomalies that could cause continuous shunting into the right atrium, such as UCS, coronary artery fistula, or partial or total anomalous pulmonary venous connection (5, 15, 16).

**Figure 3:**
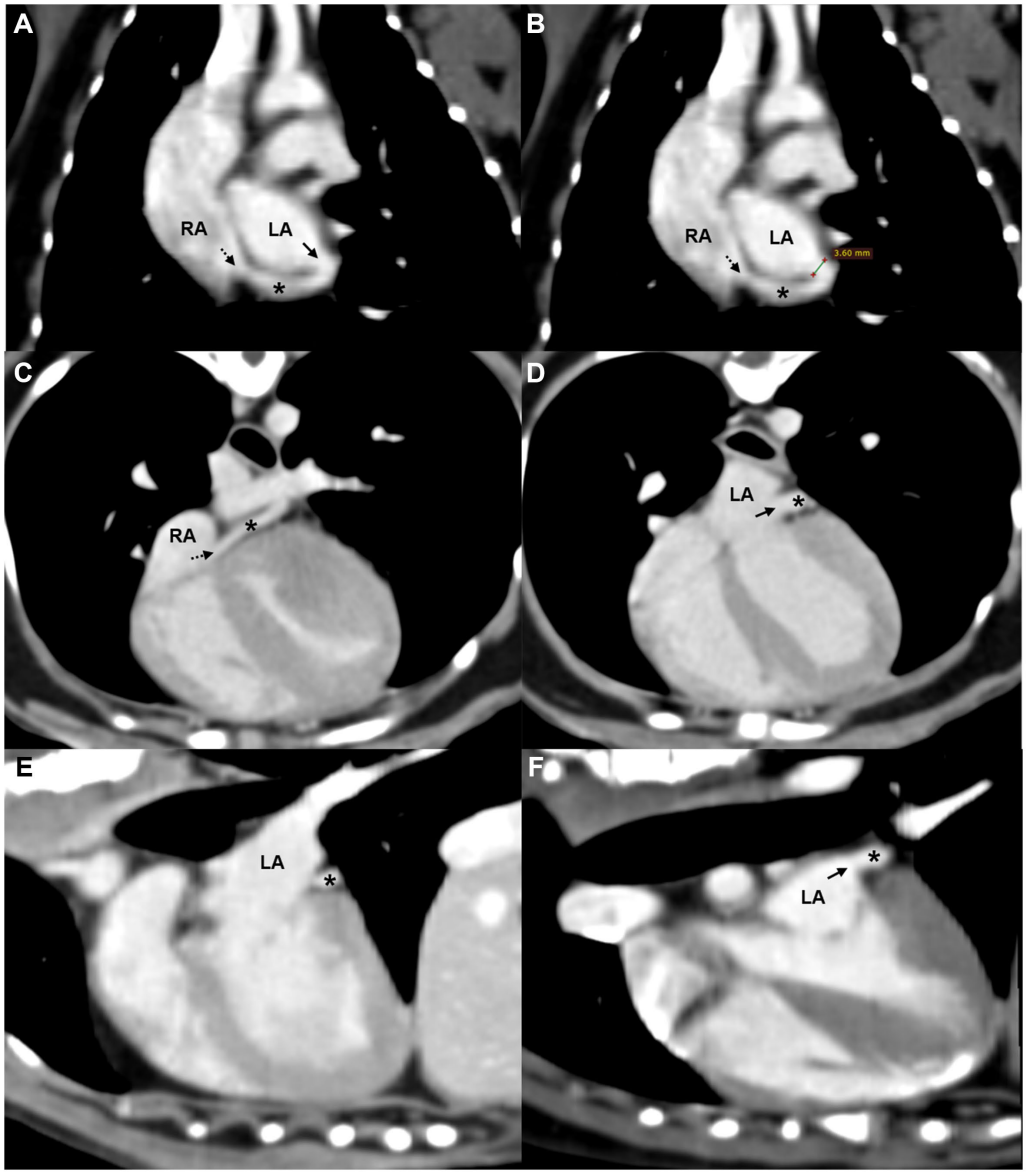
Oblique reformatted CT images of the **(A,B)** dorsal, **(C,D)** axial, and **(E,F)** sagittal planes showing a partial defect between the coronary sinus and the LA. A tubular structure consistent with the coronary sinus (asterisk) courses along the posterior aspect of the LA and drains into the RA via the coronary sinus ostium (black dashed arrow). A partial defect (black solid arrow) in the septum between the coronary sinus and LA is visible in panels **(A,D,F)**, with similar contrast enhancement noted between the two chambers. Measurement of the defect is shown in panel **(B)**. LA, left atrium; RA, right atrium; *, coronary sinus.

The CT findings confirmed a partial defect (3.6 mm) between the midportion of the coronary sinus and the left atrium (Figure 3). The coronary sinus measured 3.5 mm in diameter at both the ostium and the tubular segment. Considering that the reported mean ostium diameter in dogs is 5.5 ± 1.3 mm (17, 18), no obvious dilation was identified in this case. Contrast enhancement patterns on CT were similar in both the coronary sinus and LA, supporting the presence of a left-to-right shunt from the LA to the coronary sinus (5). No other congenital anomalies were observed. Based on these findings, the dog was diagnosed with a partial UCS. Serial echocardiographic evaluations and monitoring for clinical signs suggestive of right-sided volume overload or pulmonary hypertension were recommended to assess potential progression of the condition over time.

To investigate potential genetic factors underlying UCS in this dog, WES was conducted following previously established methods (19). WES was performed on DNA isolated from the blood of the dog by Theragen Bio (Seongnam, Republic of Korea) using the Illumina NovaSeq6000 platform (Illumina, San Diego, CA, United States). Exomes were captured using the SureSelect XT Canine All Exon V2 kit (Agilent Technologies, Santa Clara, CA, United States). Sequencing reads were aligned to the CanFam3.1 genome using the BWA-MEM (version 0.7.1). Variants were identified using SnpEff software (version 4.3). An initial set of 87,610 variants was identified, which was filtered based on annotation criteria, yielding 14,926 variants. Further filtering using public databases and literature reduced the variants to 230 (20, 21), with 13 identified as potentially pathogenic based on SIFT predictions. To evaluate the conservation and functional significance of these variants, comparisons were made with sequences from 12 reference species, including *Canis lupus familiaris*, *C. lupus dingo*, *Zalophus californianus*, *Ursus americanus*, *Lynx canadensis*, *Suricata suricatta*, *Felis catus*, and *Homo sapiens*. Cross-species analysis revealed that four candidate missense SNVs were unique to the subject and absent in other reference species.

**Figure 4:**
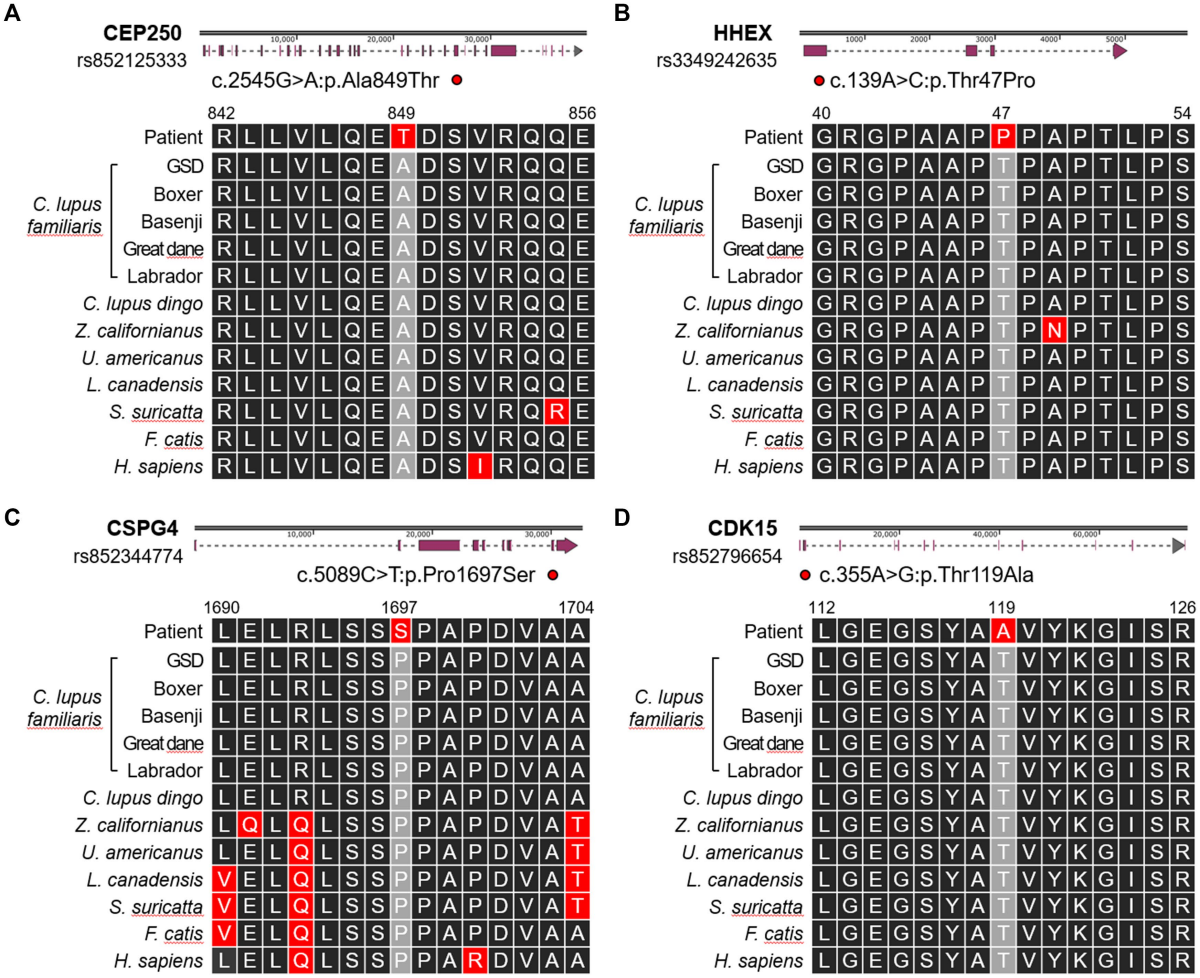
Schematic illustration showing the locations of the single-nucleotide polymorphisms present in **(A)** CEP250, **(B)** HHEX, **(C)** CSPG4, and **(D)** CDK15 genes. Altered amino acid sequences are compared with 12 references including *Canis lupus familiaris* (German Shepherd Dog, Boxer, Basenji, Great Dane, Labrador Retriever), *Canis lupus dingo* (dingo), *Zalophus californianus* (California sea lion), *Ursus americanus* (American black bear), *Lynx canadensis* (Canada lynx), *Suricata suricatta* (meerkat), *Felis catis* (domestic cat), and *Homo sapiens* (Human).

Consequently, we identified four candidate missense SNVs (Figure 4). A heterozygous SNV was present in CEP250 at position Chr24:24092656G>A (rs852125333), leading to c.2545G>A, resulting in a p.Ala849thr change. The HHEX gene had a heterozygous SNV at position Chr28:7234149A>C (rs3349242635), leading to c.139A>C, resulting in a p.Thr47Pro alteration. A heterozygous SNV was identified in CSPG4 at position Chr30:38397649G>A (rs852344774), leading to c.5089C>T, resulting in a p.Pro1697Ser change. Lastly, CDK15 had a heterozygous SNV at Chr37:11734372A>G (rs852796654), leading to c.355A>G, resulting in a p.Thr119Ala alteration. All variants were classified as missense variants with moderate predicted impact and were assessed as deleterious based on SIFT prediction scores ranging from 0.003 to 0.021.

## 3 Discussion

UCS is a rare congenital cardiac anomaly characterized by partial or complete absence of the coronary sinus roof, leading to an abnormal communication between the coronary sinus and the LA (5, 7–9). Normally, the coronary sinus drains deoxygenated blood from the myocardium into the RA (22). However, in the presence of UCS, blood shunts from the LA into the coronary sinus and then into the RA, functionally resembling an atrial septal defect. The physiological impact varies with the size of the defect; larger UCS lesions can produce significant volume overload on the right heart (5, 8, 10). In humans, UCS often coexists with other congenital cardiac anomalies, such as persistent left superior vena cava (PLSVC), atrial septal defect and endocardial cushion defect (8).

In the present case, UCS was incidentally discovered in an asymptomatic dog. Transthoracic echocardiography revealed a markedly visualized coronary sinus on Color Doppler imaging. Additionally, although no atrial septal defect was identified, continuous blood flow of undetermined origin entering the RA was observed. No cardiac remodeling or pulmonary hypertension was detected, and a bubble study ruled out a right-to-left shunt. On subsequent CT, a partial defect between the LA and the coronary sinus was identified, consistent with a diagnosis of partial UCS. No additional congenital cardiac anomalies were detected. Although ECG-gated CT was not performed, the LA and coronary sinus exhibited similar contrast enhancement patterns on the CT angiogram, suggesting the presence of a left-to-right shunt (5). Accordingly, the continuous flow entering the right atrium was considered to result from UCS.

The absence of clinical signs at the time of diagnosis was likely attributable to the small defect size and minimal shunting. This was supported by a *Q*_p_/*Q*_s_ ratio of 1.3, indicating a left-to-right shunt of hemodynamically insignificant magnitude (23). The LA/Ao ratio was 1.7, which, although within the reported normal range, was close to the upper limit (13). While slight overestimation of the LA/Ao ratio cannot be excluded, it is also possible that the calculated *Q*_p_/*Q*_s_ underestimated the actual shunt volume, particularly given the small body size of the patient (3.8 kg) and the operator-dependent nature of these measurements. Despite the absence of gross coronary sinus dilation, the prominent Color Doppler signal in the coronary sinus was interpreted as a result of increased blood flow into this structure, which may precede anatomical enlargement. Therefore, periodic echocardiographic follow-up was recommended, not only to reassess shunt volume but also to monitor for additional indicators of hemodynamic progression, such as right atrial and right ventricular dilation, progressive coronary sinus enlargement, and reversal of shunt flow. In addition, it was recommended to monitor for clinical signs of right ventricular overload or pulmonary hypertension, such as dyspnea, fatigue, palpitations, cyanosis, syncope, and peripheral edema (24).

Although transthoracic echocardiography identified continuous flow into the right atrium, the UCS defect could not be directly visualized. If 2D or 3D transesophageal echocardiography had been performed, the defect might have been detected, as transesophageal echocardiography offers better visualization of posterior cardiac structures (25). Cardiac magnetic resonance imaging can also aid in UCS diagnosis by offering detailed anatomical assessment and flow quantification via phase-contrast cine magnetic resonance imaging. Additionally, ECG-gated CT may improve detection of small defects by reducing motion artifacts (26). Such detailed assessments are particularly relevant when considering surgical or interventional repair in hemodynamically significant cases. Therefore, the potential application of these modalities in veterinary cardiology warrants further investigation, especially for rare and often underdiagnosed anomalies such as UCS.

In humans, UCS is morphologically classified based on the size and location of the defect and the presence or absence of a PLSVC, which significantly influences surgical planning and clinical management (8). UCS is commonly classified into four types based on the extent and location of the defect and the presence or absence of PLSVC: Type I (completely unroofed with PLSVC), Type II (completely unroofed without PLSVC), Type III (partially unroofed in the midportion), and Type IV (partially unroofed in the terminal portion) (27, 28). In the present case, the defect was located in the midportion of the coronary sinus without associated vascular anomalies, corresponding to Type III. A modified classification by Xie et al. (8) further divides UCS into three main types—complete absence of the coronary sinus (Type I), midportion unroofing (Type II), and terminal portion unroofing (Type III)—each with “a” (with PLSVC) and “b” (without PLSVC) subtypes. According to this scheme, the present case is classified as Type IIb. Although such classification systems have not yet been established in veterinary medicine, their application could help standardize diagnostic approaches and assist in surgical decision-making, highlighting the need for further research in this field. In veterinary medicine, UCS is only rarely diagnosed. This may reflect not only the true rarity of the condition but also a significant degree of underdiagnosis. The present case suggests that, as in humans, asymptomatic UCS can occur in dogs and emphasizes that the condition should not be overlooked even in the absence of clinical signs.

To date, only two cases of UCS have been reported in the veterinary literature. A previous case reported by Zani et al. (6) described a French Bulldog with severe pulmonary stenosis, in which angiography during right ventricular catheterization revealed anomalous venous drainage from a persistent left cranial vena cava into the left atrium. A subsequent bubble study confirmed a right-to-left shunt, as evidenced by immediate opacification of the left atrium following agitated saline injection. Although the authors did not specifically mention UCS, the direct drainage of persistent left cranial vena cava into the left atrium strongly suggests the presence of UCS type I (29). However, no anatomical confirmation or advanced imaging-based diagnosis of UCS was provided. This case underscores the importance of thorough diagnostic evaluation, including complete echocardiographic assessment and cross-sectional imaging modalities such as CT and cardiac magnetic resonance imaging, to accurately identify rare congenital anomalies such as UCS, especially in asymptomatic patients. Shin et al. (5) reported a case in a Dachshund with a larger UCS defect (15 mm) that caused pulmonary overcirculation and right ventricular volume overload, necessitating surgical intervention. In contrast, the coronary sinus defect in the present case was smaller (3.6 mm), which may account for the lack of clinical signs and cardiac remodeling. Unlike previous reports, this is the first case of UCS in a dog without clinical signs or concurrent cardiac anomalies.

In an effort to shed light on the genetic basis of UCS in dogs, we investigated four candidate genes that might contribute to UCS in a dog. UCS, a rare congenital heart defect, presents significant challenges for genetic investigation due to the limited availability of genomic data (30). While genes like NF1 and SMN2 have been implicated in congenital heart defects in humans (31, 32), no equivalent findings have been reported in other animals. To address this limitation, we utilized public databases and comparative analyses across 12 reference species, identifying SNV sites conserved in all species except the subject. This finding suggests that the identified mutations are unique to this dog and may contribute to the pathogenesis of UCS. As a future diagnostic approach, large-scale genomic analyses in a cohort of dogs diagnosed with UCS will be important for identifying shared genetic mutations and clarifying the causal relationship between these mutations and UCS development.

In the subject, we have identified four candidate missense SNVs: CEP250, HHEX, CSPG4, and CDK15. Each of these genes has been implicated, either directly or indirectly, in congenital heart defect. First, CEP250, a centrosomal protein, has been linked to left-sided congenital heart lesions through GWAS and eQTL analyses (33, 34). HHEX, a Homeobox gene family member, regulates early cardiac development and cardiomyocyte formation (35). CSPG4, a transmembrane proteoglycan, is essential for vascular development and stability (36). CDK15, a cyclin-dependent kinase involved in cell cycle regulation and tissue development, plays a role in cardiovascular development (21, 37).

The limitations of this case report are as follows. First, although regular cardiac monitoring was recommended to evaluate potential disease progression, follow-up data were not available at the time of writing; therefore, the longitudinal course of the condition could not be documented. Second, the absence of ECG-gated CT imaging may have introduced motion artifacts, potentially affecting the precision of anatomical measurements (9). Lastly, genetic analysis was restricted to a single case, limiting the statistical significance of our findings.

This report presents the first documented case of UCS without clinical signs or associated congenital anomalies in a dog, emphasizing that this rare condition should not be overlooked even in asymptomatic cases. Additionally, four candidate genes potentially associated with UCS are reported, providing a foundation for further genetic investigations into this rare condition. Further research and multi-institutional cooperation are needed to clarify the genetic mechanisms of UCS and establish evidence-based guidelines for its diagnosis in veterinary medicine.

## Data Availability

The Whole Exome Sequencing (WES) raw data generated and analyzed in this study have been deposited in the NCBI Sequence Read Archive (SRA) and are publicly available. The data can be accessed using the following accession numbers: BioProject PRJNA1294427, BioSample SAMN50096542, and SRA SRR34653185 via the direct link https://www.ncbi.nlm.nih.gov/sra/SRR34653185.
